# Lessons from a large-scale COVID-19 vaccine trial

**DOI:** 10.1172/JCI163202

**Published:** 2022-09-15

**Authors:** Ranjeet Singh Mahla, Lynn B. Dustin

**Affiliations:** Kennedy Institute of Rheumatology, University of Oxford, Oxford, United Kingdom.

## Abstract

Global vaccination coverage remains indispensable in combatting the ongoing SARS-CoV-2 pandemic. Safety, efficacy, and durability of immune protection are the key parameters of randomized controlled trials (RCTs) and are essential for vaccine approvals, global distribution, and comprehensive population-vaccination programs. Immune protection from either vaccination or natural infection decreases over time, further challenged by rapid viral evolution. In this issue of the *JCI*, Sobieszczyk and colleagues report an update on the safety, efficacy, and durability of immune protection of AZD12222 in a large-scale, multinational, Phase III RCT. They report that protection lasted through 6 months, with immunity waning after 180 days. The study also highlights challenges facing vaccine trials, including the need for early unblinding for vulnerable participants, which may affect outcome measurements. Another challenge is to ensure fair representation of marginalized and minority ethnic groups in vaccine safety and efficacy studies worldwide.

In this issue of the *JCI*, Sobieszczyk et al. report updated results from a large-scale, multinational trial of the SARS-CoV-2 vaccine, AZD12222, in the United States, Peru, and Chile. Participants were followed for at least 180 days after their last vaccine dose. Neutralizing antibody titers increased over the first 57 days and declined in all groups by 180 days after vaccination. The study confirmed the safety and efficacy of this vaccine and also highlights emerging challenges of large-scale SARS-CoV-2 vaccine trials more than 2 years into the pandemic (1).

## RCT safety and efficacy

The World Health Organization (WHO) sets safety and efficacy as critical measurable parameters for all vaccines undergoing randomized controlled trials (RCTs). Following a complete course of vaccination, COVID-19 vaccines should persistently demonstrate high effectiveness against SARS-CoV-2 infection and minimize fatal outcomes of the disease. Currently, 40 COVID-19 vaccines are approved and distributed in at least in 1 country (https://covid19.trackvaccines.org). The complete course of approved vaccines or vaccines in different stages of RCT may comprise single or multiple doses. Approved vaccines target either viral components (subunit vaccines) or the whole virus. Component vaccines present viral antigens as purified proteins or virus-like particles encoded in DNA or RNA or via replicating or nonreplicating viral vectors. Whole-virus vaccines utilize either inactivated or live attenuated SARS CoV-2. The nonreplicating viral–vectored vaccine AZD1222 (ChAdOx1 nCoV-19; also known as Vaxzevria) is now approved in 141 countries. The safety and efficacy of AZD1222 are continuously monitored, reported, and validated across multiple completed and ongoing RCTs (2, 3). Widely distributed COVID-19 vaccines (4–8) are proven safe and effective.

## Long-lasting immune protection and cross-protection

**Figure 1:**
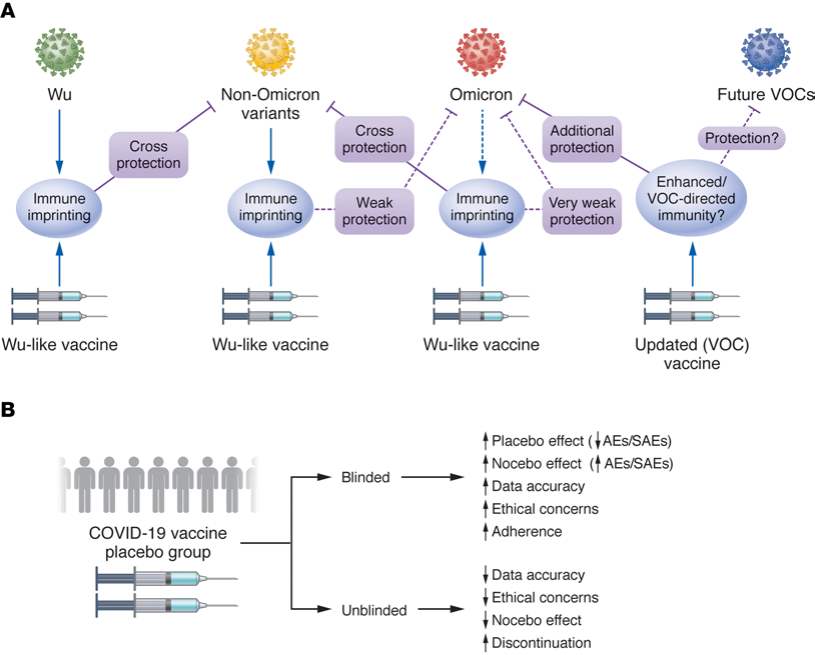
Lessons and questions from a large-scale COVID-19 vaccine trial. (**A**) Immune imprinting and durability of protection. The immunogenic components in SARS-CoV-2 vaccines are derived from early (2019 to 2020) WT or Wuhan-like (Wu-like) viral isolates. Vaccine-stimulated immunity results in antibodies that neutralize infection by homologous or closely related viral strains (solid lines), but does not confer long-lasting protection. Immune imprinting from either vaccination or infection with early SARS-CoV-2 strains means that subsequent infections with newer SARS-CoV-2 variants stimulate existing memory cells specific for previously encountered SARS-CoV-2 variants, rather than recruiting additional immune cells better suited to the new variants. Therefore, the antibodies produced may not efficiently neutralize subsequent variants (dashed lines). Over time, protection and cross-protection are further diminished due to declining antibody levels. The Omicron variant and emerging lineages bear numerous immune escape mutations and are poorly neutralized by antisera specific for older SARS-CoV-2 strains. The durability and breadth of immune protection may be enhanced with booster dose vaccination and updated vaccines. (**B**) Trade-offs of placebo controls. Placebo control blinding and unblinding are a trade-off between the accuracy of RCTs’ primary and secondary outcome measures and ethical/social justice concerns.

Vaccine- or natural infection-acquired immunity to respiratory viruses declines over time (9). Therefore, it is increasingly important to evaluate the durability of immune protection against circulating and emerging strains of SARS-CoV-2 (4–8, 10–13). Cellular and humoral immunity following natural SARS-CoV-2 infection may persist for more than 1 year (10) and immune imprinting by immunization or natural infection may affect subsequent responses to emerging strains and variants of concern (VOCs) (10, 14) (Figure 1A). Thus, preexisting immunity to early SARS-CoV-2 strains may interfere with immune priming against new SARS-CoV-2 strains, perhaps because of biased recruitment of immune memory cells specific for the previous strain. This phenomenon is also known as “original antigenic sin.” Furthermore, antibodies specific for earlier strains may have low affinity for emerging variants. Natural infection–acquired immunity may not protect against emerging virus strains but can alter the durability of immune protection following vaccination (10). The combined effects of immune imprinting, natural waning of antibody titers, and rapid viral evolution may all contribute to substantially reduced vaccine efficacy against divergent viral strains such as Omicron (B.1.1.529) and its descendent lineages. Immune imprinting following SARS-CoV-2 B.1.1.7 (alpha) infection can reduce the durability of protection against Omicron following BNT162b2 vaccination (14). In contrast, Omicron infection following 3 doses of BNT162b2 vaccine can confer durable cross-protection against earlier VOCs (14). AZD12222, BNT162b2, and mRNA-1273 are among the most widely distributed vaccines. In a small-scale trial of the mRNA-1273 vaccine, a high neutralizing antibody titer persisted through 6 months after administration of the second dose (4). The durability of vaccine efficacy can also be estimated by regression analysis of public health data sets (15); for example, in North Carolina, immune protection from mRNA-1273 and BNT162b2 vaccines lasted 7 months (11). As reported by Sobieszczyk and colleagues (1), a small number of infections with contemporaneous VOCs were observed in the vaccinated and placebo groups within the first 6 months after immunization; data collection was completed before the emergence of the Omicron strain.

In the current study, Sobieszczyk and colleagues update the results of an ongoing large-scale, multi-national, placebo-controlled, Phase III RCT of AZD12222 in the United States, Peru, and Chile (1). The trial, which included 32,450 participants, had a primary efficacy endpoint of symptomatic SARS-CoV-2 infection, while secondary endpoints included disease severity, infections per number of subjects who seroconverted post vaccination, and emergency visits. Here, participants were followed for 6 months after their first vaccine dose. This allowed assessment of the durability of immune protection and of changes in neutralizing antibody levels over time. Many participants were considered eligible to be unblinded and received a nonstudy COVID-19 vaccine before the end of the study (Figure 1B). Vaccine efficacy measures were stratified by age, sex at birth, race, ethnicity, BMI, comorbidities, OSHA risk categories, and participants’ global regions of origin for the period of double-blinding (days after second vaccine dose) until receipt of nonstudy COVID-19 vaccine. The AZD12222 vaccine was 67% and 95.7% effective in preventing symptomatic SARS-CoV-2 infection and severe illness during the double-blinding period, respectively. Overall, the AZD12222 vaccine durability of immune protection through 6 months is found to be 70.2% effective. There were no concerning safety issues, and most recorded adverse events (AEs) were mild to moderate. Irrespective of age, sex, comorbidities, ethnicity, or other risk covariates, AZD12222 elicited a robust humoral response through 6 months, with antibody titers declining after 180 days. Waning of immunity was relatively low among individuals seropositive for SARS-CoV-2 at baseline compared with those who were SARS-CoV-2-seronegative.

## Complexities of placebo controls

The study highlights some of the challenges posed by double-blinded, placebo-controlled vaccine trials. There are trade-offs between ethical concerns and the quality of primary and secondary outcome measures (16). Many participants were in elevated-risk groups, becoming eligible for nonstudy vaccines during the study period. Serologic studies revealed that some participants in the placebo-group appeared to have received a vaccine off-study. Additionally, adverse events (AEs) and severe adverse events (SAEs) were recorded in the placebo group; the phenomenon is known as the nocebo effect (Figure 1B). Systematic review and meta-analysis of RCTs show that the nocebo effect contributes to 76% and 52% of AEs after the first and second dose of COVID-19 vaccines, respectively (17).

## Disparities and challenges

There are disparities in COVID-19 RCT enrolment representation and vaccine coverage, mainly at the level of geographical representation, income groups, and inclusion of minority ethnic groups and persons of color (2, 18, 19). Globally, 12.29 billion COVID-19 vaccine doses have been administered; however, they have disproportionately benefited people from high-income countries, and only 19.6% of people in low-income countries have received at least 1 dose (18). The completed and ongoing SARS-CoV-2 vaccine RCTs in Africa are largely focused on South Africa, Kenya, and Egypt (https://covid19.trackvaccines.org/trials-vaccines-by-country/#trials) (20). Disparities in trial enrolment and vaccine coverage can be attributed to a number of underlying factors, including, but not limited to, vaccine storage requirements, supply chain management, healthcare logistics, complex protocols, policy-driven approvals, and distrust of medical advances. Few RCTs or healthcare records on primary and secondary outcome measures are stratified to patient origin, ethnicity, and geographical region (19). Uneven RCT distribution and vaccination coverage may hamper accurate determination of vaccine efficacy. More attention should be paid to ensuring that RCTs include and benefit peoples of diverse ethnicities, indigenous populations, and isolated tribal communities.

## Going global and the next steps

The most effective vaccines will provide long-term immune protection for populations of diverse origins and ethnicities. Centrally managed clinical trial platforms such as the WHO’s Solidarity Trial, the University of Oxford’s RECOVERY, the NIH’s ACTIV initiative, and others (21, 22) are working on expanding RCTs to multiple countries, to deal with emerging VOCs and to inform decision making on vaccination access, therapy, and boosting strategies. Disparities can be minimized through corrections at the steps of vaccine production, fair allocation, affordability, and deployment of RCTs and vaccine distribution through central platforms (23). The WHO’s COVAX platform aims to achieve equitable development, production, and distribution of COVID-19 resources worldwide. For equitable access and diversified recruitment, remote clinical trials with social media advertisement can be deployed in developed countries where disparities can be attributed to hesitation and resource allocation among ethnic minorities (24). The European Medicine Agency’s revised guidelines on COVID-19 vaccine trials also recommend remote data acquisition and verification strategies (25). Deploying digital technologies and integrating on-site, remote, and virtual monitoring systems can minimize commute inconvenience and hesitancy among participants. Such technology-driven management processes can speed up recruitment and increase trial adherence. Compared with the conventional clinic-based approach, remote clinical trials are still far from routine implementation. In the coming years, large-scale RCTs will be more technology-driven.

## Version 1 09/15/2022

Electronic publication
